# FedMSA: A Model Selection and Adaptation System for Federated Learning

**DOI:** 10.3390/s22197244

**Published:** 2022-09-24

**Authors:** Rui Sun, Yinhao Li, Tejal Shah, Ringo W. H. Sham, Tomasz Szydlo, Bin Qian, Dhaval Thakker, Rajiv Ranjan

**Affiliations:** 1 School of Computing, Newcastle University, Newcastle upon Tyne NE1 7RU, UK; 2 Institute of Computer Science, AGH University of Science and Technology, 30-059 Krakow, Poland; 3 Department of Computer Science, University of Bradford, Bradford BD7 1DP, UK

**Keywords:** federated learning, model selection, device adaptation, model adaptation, orchestration, distributed system

## Abstract

*Federated Learning* (FL) enables multiple clients to train a shared model collaboratively without sharing any personal data. However, selecting a model and adapting it quickly to meet user expectations in a large-scale FL application with heterogeneous devices is challenging. In this paper, we propose *a model selection and adaptation system for Federated Learning* (FedMSA), which includes a hardware-aware model selection algorithm that trades-off model training efficiency and model performance base on FL developers’ expectation. Meanwhile, considering the expected model should be achieved by dynamic model adaptation, FedMSA supports full automation in building and deployment of the FL task to different hardware at scale. Experiments on benchmark and real-world datasets demonstrate the effectiveness of the model selection algorithm of FedMSA in real devices (e.g., Raspberry Pi and Jetson nano).

## 1. Introduction

Cloud computing has positively affected people’s daily life and industrial production since it was proposed around 2005 [1], such as social media, cloud storage services and industry 4.0. Meanwhile, the *Internet of Things*  (IoT) industry is developing rapidly, and the number of IoT devices worldwide will reach more than 41 billion by 2027, which is five times that of about 8 billion in 2019, as forecasted by Business Insider [2]. Billions of IoT devices connect to the Internet to produce large volumes of data daily, which are transferred and stored in cloud servers. However, the challenges of complying with rigorous data protection regulations such as EU/UK *General Data Protection Regulation* (GDPR) [3] make it extremely difficult to collect data across heterogeneous sources. Besides, centralized data storage, unified servicing and modelling in the cloud are also facing massive pressures from the constraints of network bandwidth of IoT devices and users’ privacy leakage problem [4]. As a consequence, *Machine Learning* (ML)-applied device intelligence becomes vital and important development direction of IoT. The emergence of on-device learning solves the problem of bandwidth and privacy by directly using local data to train ML models and make predictions instead of using the predominant paradigm of ML that trains a model in the cloud and inferences locally [5].

**Figure 1:**
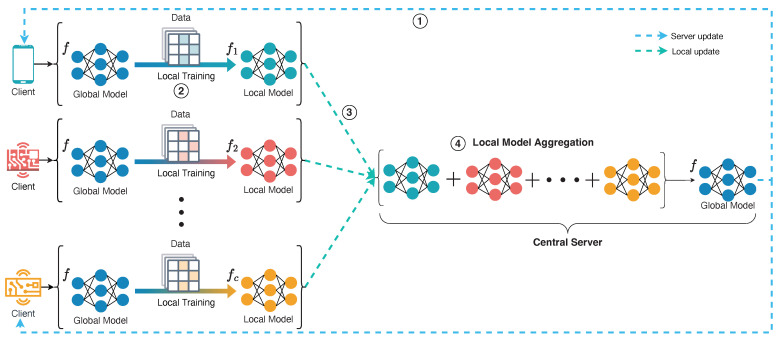
Vanilla Federated Learning Workflow.

However, because of the enormous difference in data distribution and diversity, the on-device learning may lead to a serious problem in that the model trained locally cannot achieve the same performance as the model trained centralized in the cloud. To cope with the aforementioned challenge, *Federated learning* (FL) was proposed to let different clients (e.g., mobiles, servers or companies) collaboratively train a unified model while keeping the training data locally [6,7]. FL has been applied to various privacy-critical scenarios such as hospitals [8], banks [9] and autonomous vehicles [10]. In FL, sharing raw personal data is strictly prohibited, and only model parameters can be transmitted between the server and clients. In this way, the local model performance of all clients could be improved while ensuring privacy security. One round model training process of horizontal FL is shown in Figure 1: ① A central server broadcasts the global model to all clients; ② all clients train the latest global model using their local data to get the latest local model; ③ all clients then upload their latest local model to the central server; ④ finally, the central server aggregates all local models from clients to update the global model. Since the aggregation progress will happen after receiving all local trained models in horizontal federated learning, the overall system training time is determined by the slowest client. To address this problem, authors in [11] proposed an adaptive deadline determination algorithm for mobile device selection. Nevertheless, it effectively reduces the FL system’s training time but does not trade-off both training efficiency and final model performance.

Based on [6], the FL setting could be divided into “cross-silo” and “cross-device” regarding training among different organizations or devices. The focus of this paper is on cross-device FL, which means the clients include a large number of mobile or IoT devices. Selection and adaptation of the task model structure is a significant challenge for cross-device FL with heterogeneous hardware. Historically, most FL research has directly used the widely adopted model structure in centralised machine learning [7] or undertook manual designing of the structure [12] based on the target training task. More recently, several studies have started to focus on using *Neural Architecture Search* (NAS) [13] to automatically generate an optimal model structure. They mainly focus on optimising model performance for the reference stage, such as higher accuracy and lower inference latency. In practice, this may hinder the applicability of FL since model training efficiency and hardware heterogeneity also affect the efficiency and performance of the whole FL system. Thus, FL developers find it difficult to achieve the expected results without cross-platform model adaptation and training and inference running simultaneously.

To improve the applicability of practical FL, we consider a realistic setting in which FL system contains heterogeneous hardware where some clients contribute to the central model and use the local model to make predictions simultaneously. Our goal is to select an expected model structure that trades-off the training efficiency (e.g., training time, network utility) and model performance (e.g., accuracy, inference latency), while considering the developer’s choices. For example, some developers are more sensitive to model training efficiency, and some want to get a model with optimal performance. Because NAS requires high computational demand, some methods need thousands of GPU days to search the best architecture for some tasks, which can be an unacceptable and catastrophic situation for developers [14]. So our work is based on the pre-searched models’ metrics from NAS-Bench-201 [15] and HW-NAS-Bench [16]. Nevertheless, although our model selection algorithm can accurately select the optimal model structure under the given parameter, we recognized that it is difficult to accurately select the optimal model structure that meets the developer’s expectations through our model algorithm by one-time deployment as FL developers may have more personalized preferences. Therefore, we need a system that supports rapid task deployment at scale, iterating over the results to get the optimal result that developers expect.

In this paper, we propose *FedMSA*, *a **M**odel **S**election and **A**daptation system for **Fed**erated Learning*, which can address the previously listed challenges. It reduces the complexity of FL system deployment by providing automation of adaptation and deployment of training task as microservices in a FL life-cycle along with *model selection* (MS) algorithm. Our MS algorithm could help FL developers search for an optimal model structure based on their expectations in training efficiency and final model performance. On comparing the results of our MS algorithm with manual design and direct selection of a model by one factor from NAS, the model selected by our algorithm achieves good performance in training efficiency and model performance. Similar ideas have been proposed in network speed optimization; for example, the MARS method proposed in [17] maintains balance for comprehensive utilization, transmission efficiency, and monetary cost to optimize high-speed mobile networks. But, to the best of our knowledge, this work is the first attempt to tackle the challenges of training efficiency and model performance by identifying the optimal model structure that meets developer expectations.

Our contributions are summarized as follows:

- Methodologically, we propose a model selection algorithm.
- Practically, we design and implement FedMSA system in real distributed devices with heterogeneous hardware.
- Empirically, we demonstrate the effectiveness of MS algorithm on real-world datasets and compare with other methods.

The paper is organized as follows. Section 2 deals with an overview of related works and state-of-the-art studies. Section 3 discusses the motivation, while Section 4 describes the proposed algorithm and the system. Section 5 presents the evaluation of our algorithm. Finally, the paper is summarized, and future work is discussed.

## 2. Background and Related Work

Machine Learning (ML) was proposed in 1959 [18], as a part of *artificial intelligence* (AI). ML imitates human learning by modelling experience (historical data) and can constantly self-iterate to improve its performance. During the past two decades, ML made significant progress by successful application in many areas of technology and science, for example, computer vision, natural language processing, autonomous vehicle and robotics [19]. As for the traditional ML tasks, such as classification or linear regression, the user only needs to spend several minutes or hours training the model on a server or personal computer. *Deep learning* (DL) made the number of technological breakthroughs after deep conventional neural networks were proposed in 2012 [20] and significantly outperformed ML. Meanwhile, the number of layers of models designed by ML engineers is increasing, such as ResNet [21]. ResNet allows the network layers of *neural network* (NN) to be continuously superimposed without negatively affecting the model’s accuracy, so the authors of ResNet tried increasing it to 110 layers with 1.7 million parameters. In addition, the model structure continues to become increasingly complex, such as the Transformer [22] where the number of parameters of the basic Transformer model is 67 million, and the big Transformer model could achieve 213 million. Both models have more computation complexity and require more computation resources than the three layers *multi-layer perception* (MLP) model widely used in classic ML tasks.

On the other hand, with an increasing number of edge and IoT devices being connected to the Internet, high volumes of data are being generated and transmitted to the cloud server to train a model. However, the simultaneous and high-frequency transmission of a large amount of data can cause network overload and leak user privacy. Furthermore, data regulations (such as the GDPR) make it hard to access cloud services providers or collect data from terminal devices (e.g., mobiles, sensors). In 2016, the authors in [7] proposed FL, which is a solid solution to the above problems. It lets clients train a central model by feeding their own data collaboratively. Compared with traditional ML model training, sharing raw personal data in FL is strictly prohibited, and only model parameters are transmitted between the server and clients. While protecting user privacy and network traffic, the training model of FL could learn the representation of every client’s local dataset to improve model performance. However, similar to the classic distributed system, hardware heterogeneity in cross-device FL can also bring many challenges such as how to generate a model that can be trained efficiently and perform well on heterogeneous hardware.

In centralized deep learning, the developer usually manually designs a model structure for the target task based on their knowledge or experience, such as ResNet [21] in the image classification task, and Transformer [22] and Bert [23] in machine translation task. Manual design of model structures not only increases the labour and time costs of model design and testing, but also makes it difficult for machine learning engineers to design models with good performance in a short period of time. As a consequence, numerous different NN connections choices have to be tried [24]. To avoid this, in recent years, some studies have started to use *neural architecture search* (NAS) instead of manually generated network models. NAS aims to find good architectures, and NAS methods outperform manually designed architectures on some tasks such as object detection [25,26] or semantic segmentation [27,28,29]. After the NAS first-time proposed in [24], most of studies focused on searching for a model structure with the highest accuracy [30]. As the accuracy of model architecture has improved and becomes state-of-art, some works have started to consider how to strike a balance between performance and efficiency [29,31]. However, due to user data privacy security and network traffic pressure, centralized ML in cloud is shifting to on-device learning and inference on target devices. Thus, some studies involve searching of a model architecture by considering target hardware [15,32,33,34] or design a specific NAS for target hardware such as [31,35] employ NAS for *microcontrollers* (MCU). Unlike unified model training, on-device learning may lead the model to underperform in some tasks due to lack of data diversity.

To tackle this problem, FedAvg as vanilla federated learning paradigm proposed in [7] allows thousands of clients to train a central model. As a distributed learning system, selecting a model structure is very difficult. It should consider model training efficiency in local devices, whole distributed system utility, and model performance in heterogeneous hardware. Existing NAS methods can only search for the model with best performance [25,26,27,28,29,31,35] or list all model structures and give pre-measure metrics [15,16,34], but lack a model selection method for distributed and federated machine learning systems. However, there are some works on model selection methods that have been attempted. In [36], authors proposed an inference model selection scheme which considers the desired accuracy and inference time for a single target embedded deep learning platform (e.g., Jetson Tx2). Authors in [37] aim to select a model from a large number of available source models that can maximize the predictive performance in the target domain of transfer learning, and they select a model by sorting source models according to their *mean silhouette coefficient* (MSC) score which is calculated by using cosine distance metric. Although these methods can select the optimal model, they lack consideration of model training efficiency, model performance and heterogeneous hardware for FL system. Moreover, in a real federation learning system, rapid model adaptation and model selection are equally important, FL developers are likely to select and redeploy the selected model multiple times to get the model they expect. This iterative process can be time-consuming and hence, designing a model adaptation orchestration system at scale to support rapid model selection is necessary. Although there have been various platforms supporting deployment and development of FL such as Flower [38], FATE [39], FedML [40], these platforms failed to consider heterogeneous hardware and model selection in real-world distributed systems. To the best of our knowledge, runtime model selection and adaptation system have not been performed in FL applications.

## 3. Motivation

**Figure 2:**
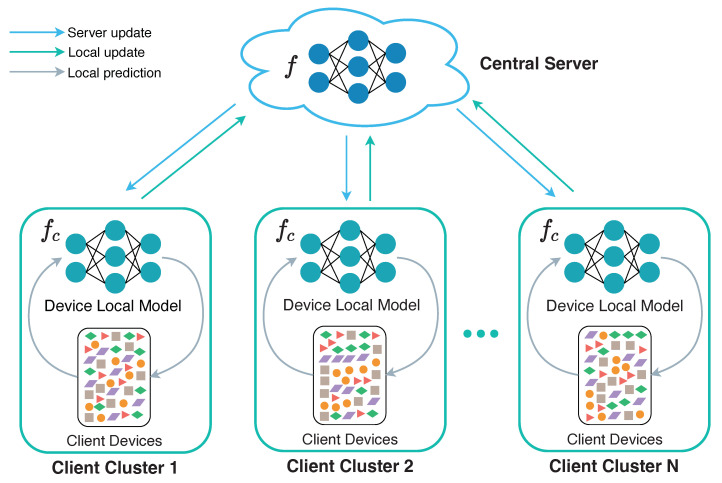
Illustration of the motivations. The client clusters represent companies where each cluster contains many heterogeneous devices as Federated Learning clients. The local model of a device participates in FL training and task inference simultaneously.

Consider a system of a real-world large-scale FL application shown in Figure 2. Edge-IoT client devices with various hardware architectures simultaneously perform local prediction tasks which contribute to a central ML model training in a cross-device FL framework. In a networked, distributed and cross-device machine learning system, the primary challenge is model design or model selection as many factors, such as local and global training efficiency, network pressure and model performance need to be considered in heterogeneous hardware architectures. As regards efficiency-sensitive model training tasks, such as recommendation systems, the cold-start phase requires the model training to achieve high accuracy within certain time constraint when a new user or item joins the network. Moreover, some performance-sensitive model training tasks, such as CCTV anomaly detection system, real-time temperature monitoring and alert system, require the model training to perform high accuracy along with low inference latency. These factors raise challenges in manual model design or model selection. Although there exists research that focuses on model selection method [36,37], they fail to consider federated and distributed machine learning framework. Model structure selection automation is crucial in balancing model training efficiency and performance.

However, the generated model may not meet the FL developer’s expectations. Therefore, a better way is to allow users define the satisfying proportion of model performance and training efficiency. Additionally, several existing NAS studies [15,32,33,34] provide model structure search space with pre-measured metrics and inference latency in various platforms (e.g., Raspberry Pi, Edge GPU device, and TPU). Nonetheless, no existing studies focus on searching for a ML model based on the developer’s preference to balance training efficiency and model performance in federated learning. A system that provides model selection and multi-platform adaptation service, and simplifies the deployment of FL tasks in real devices is a fundamental requirement.

## 4. Proposed System

This section presents the proposed FedMSA system, large-scale FL, as well as the model selection algorithms.

### 4.1. System Architecture

**Figure 3:**
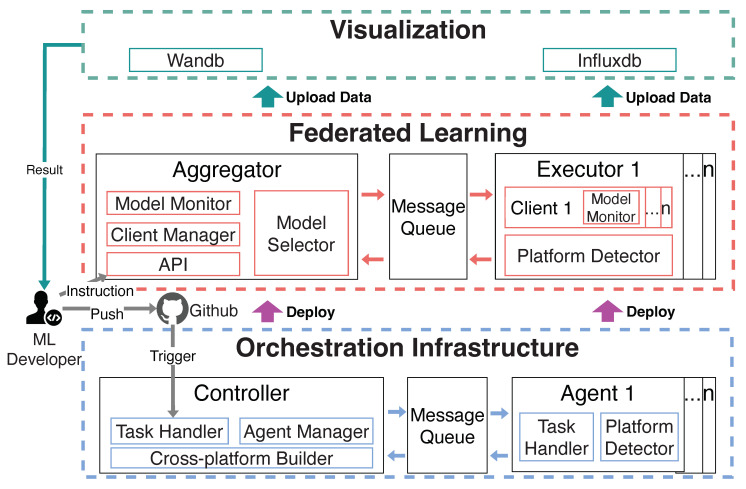
System Architecture.

In response to the previously mentioned challenges, we developed *FedMSA*, a cross-platform model selection and adaptation system for federated learning applications. We present the overview and architecture of *FedMSA* in Figure 3, which consists of three main components: *Orchestration Infrastructure*, *Federated Learning* and *Visualization*. *FedMSA* meets aforementioned requirements, providing flexibility and reliability for FL developers in personalized FL tasks deployment with automatic model selection and adaptation for a specific client’s hardware platform.

**Orchestration Infrastructure.** Automatic, rapid and agile deployment of FL task is the key to achieving effective model adaptation for a distributed system across different devices with heterogeneous hardware architecture, especially for a large-scale networked ML system. Therefore, we designed an orchestrator as the infrastructure of *FedMSA*, which supports all aforementioned functionalities from source code auto-building to auto-deployment. It consists of two main components: *controller* and *agents* running at the cloud tier and edge tier of edge-cloud computing environment respectively. Meanwhile, all messages transferring between components are handled by a message queue service, and all agents are organized by the agent manager. The training task starts after a machine learning developer pushes the latest code to Github and triggers the task handler to process the task code by calling a cross-platform builder to build FL task code into the microservices base on the hardware architecture information (e.g., x86_64, aarch64 and armv7l) of every agent host which is reported by platform detector.

**Federated Learning.** An FL system (e.g., FedAvg [7]) usually consists of thousands of participating heterogeneous devices. The scalability requirement of FL deployment necessitates a model selector to generate model structure automatically based on the developer’s expectation. Accordingly, we design a Neural Architecture Search (NAS) based model selection algorithm that traded-off model training efficiency and model performance on the heterogeneous hardware, which we discuss in detail in Section 4.2. Further, aggregator and executors are deployed by orchestration infrastructure while message queue handles communication between them where each executor deploys one/multiple clients based on the number of computation units (e.g., CPU, GPU) in the target device. A global client manager manages all clients in the aggregator including client registration, join or quit training requests, and client selection algorithm management.

Furthermore, we provide two ways for fast model adaptation: 1. developers can send an instruction to switch the current training model by the public API whereby the aggregator and all clients will stop current training tasks and load new model to execute by received commands. 2. developers can modify a parameter in the configuration file to calculate new scores for all model candidates and once the latest codes are pushed, the orchestration infrastructure will redeploy all components automatically.

**Visualization.** All model training information, model performance are uploaded to visualization tools in real-time by model monitor of Federated Learning of FedMSA. Our system supports online monitoring tool Wandb (https://wandb.ai/site accessed on 18 August 2022) and local visualization tool Influxdb (https://www.influxdata.com/ accessed on 18 August 2022).

### 4.2. Model Selection Algorithm

As mentioned in Section 1, all model structures *x* that our algorithm uses are from the search space of NAS-201-Bench *X* (the *i*th model structure is $x^{\left(i\right)}\in X$), essential metrics of every model are from NAS-201-Bench, and metrics about model inference latency in different hardware are from HW-NAS-Bench. We propose a method in Algorithm 1 to collect and organize the metrics from both NAS benchmark studies. The platform detector component in the FL to collects platform information *P* and bandwidth *B* from all clients *C* Figure 3. In particular, our system allows FL developers to pre-filter out the model with unexpected accuracy according to given set model accuracy expectation parameter α∈[0,100] in the configuration file while abnormal model structures are filtered out by system. Besides, to ensure that the impact of each metric on the final score calculation is similar, we adopt the data Min-max normalization [41] that reorganizes the distribution space of all metrics belonging to the values between 0 and 1, and is shown as:

(1) $$f\left(metrics\right)=\frac{x_{i,j}-x_{j}^{min}}{x_{j}^{max}-x_{j}^{min}}$$

In the Equation (1), *j* is the index of attribute of models (e.g,. size of model, estimated accuracy of model), *i* is the index of a model, $x_{i,j}$ represents the exact value of attribute *j* of the model *i*, and $x_{j}^{min}$ and $x_{j}^{max}$ is the minimum and maximum value of attribute *j* respectively.
**Algorithm 1:** Pseudocode for model metrics collection
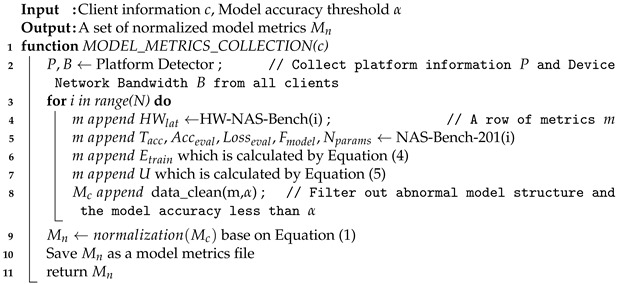


In the system design view, we consider the running memory requirements of NAS-201-Bench to be around 25G. Accordingly, in the Algorithm 1, we use one-time data collecting or pre-collecting and loading from NAS-201-Bench in the future method to save $M_{n}$ as a model metrics file. This approach not only dramatically reduces the spatial complexity of our system, but also enables our system to support more devices.

Our model score calculation algorithm mainly follows the Equation (2), and working details shown in Algorithm 2. It aims to trade-off model training efficiency and model performance base on a FL developer’s input parameter γ∈[0,1] which indicates the extent to which the developer is concerned about the efficiency of model training.

(2) Score=f(ModelTrainingEfficiency)×γ+f(ModelPerformance)×(1−γ)

**Algorithm 2:** Pseudocode for score calculating

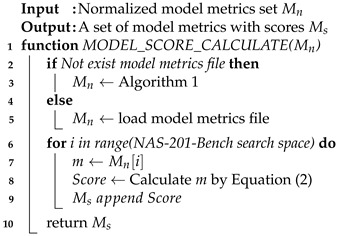



Based on the model metrics $M_{n}$ from Algorithm 1, we found that five factors can form Equation (3) to calculate model training efficiency: training time to the model accuracy $T_{train}$, FLOPs of the model $F_{model}$, numbers of parameters of the model $N_{params}$, training efficiency in accuracy increasing $E_{train}$, and bandwidth utility of a device *U*. Since some metrics are negatively correlated with model training efficiency, we set their value as negative and upon averaging, we get:

(3) $$f\left(ModelTrainingEfficiency\right)=\bar{-(T_{train}+F_{model}+N_{params}+E_{train})-U}$$

where $E_{train}$ is calculated by $T_{train}$ and expected final evaluation accuracy of the model $Acc_{eval}$ which is:

(4) $$E_{train}=\frac{T_{train}}{Acc_{eval}}$$

and *U* calculated by the average of all bandwidth of client devices $B_{c}$, and the $S_{bit}$ which means the storage bit of the development platform. In this work, we use Pytorch (https://pytorch.org/ accessed on 18 August 2022) as our development platform, so this parameter is equal to 32 bit.

(5) $$U=\frac{\bar{\sum_{c=1}^{C}B_{c}}}{N_{params}*S_{bit}}$$

We can then summarize the equation for the model training efficiency calculation as:

(6) $$f\left(ModelTrainingEfficiency\right)=\bar{-\left(T_{train}+F_{model}+N_{params}\right)+\frac{T_{train}}{acc_{eval}}+\frac{\bar{\sum_{c=1}^{C}B_{c}}}{N_{params}*S_{bit}}}$$

In order to let every metric have the same impact for score calculating, we set the calculation of $E_{train}$ and *U* before the data normalization process as shown in Algorithm 1.

As for the model performance calculating, we mainly focus on the impact of model evaluation accuracy and loss and the model inference latency in every FL client device, which is negatively correlated with model performance. It can be described as:

(7) $$f\left(ModelPerformance\right)=\bar{acc_{eval}+loss_{eval}-\bar{\sum_{n=1}^{N}Lat_{n}}}$$

Overall, the model score calculation equation can be summarized as Equation (8). We calculate scores for all model structures and wrap them up into a set $M_{s}$ for further model selection decision.

(8) $$g\left(Score\right)=\underset{ModelTrainingEfficiency}{\underset{︸}{\bar{-\left(T_{train}+-F_{model}+-N_{params}\right)+\frac{T_{train}}{acc_{eval}}+\frac{\bar{\sum_{c=1}^{C}B_{c}}}{N_{params}*S_{bit}}}}}\times \gamma +\underset{ModelPerformance}{\underset{︸}{\bar{acc_{eval}+loss_{eval}-\bar{\sum_{n=1}^{N}Lat_{n}}}}}\times \left(1-\gamma \right)$$

After defining the necessary functions, we start primary federated learning training as presented in Algorithm 3. The task starts from procedure D, and our system directly starts training if FL developer has already assigned a specific model index idx in the configuration file. If not, the system will select the model index with the highest score from the ranked model metrics list $M_{r}$.

During model training, the public API allows the developer to decide whether the current training needs to be terminated and the model adapted from the results of the monitoring data visualization tool shown in Figure 3 by sending instructions at any time. In this way, the cost of task redeployment can be significantly reduced and the developer can re-score all model structures by modifying the γ in the configuration file.
**Algorithm 3:** Algorithm of FedMSA system
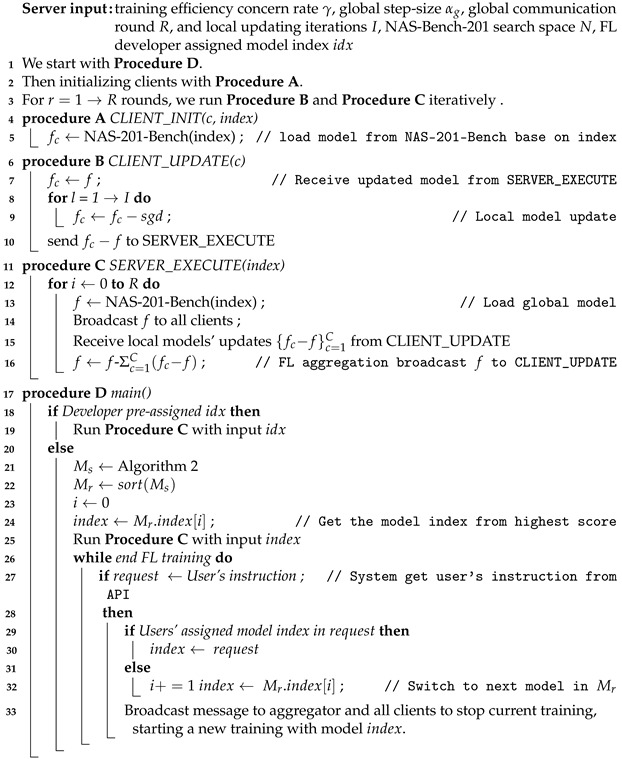


## 5. Evaluation

In this section, we present the results of the experiments performed with proposed FedMSA system. We also discuss the technical details of the experimental testbed.

### 5.1. Experiment

**Figure 4:**
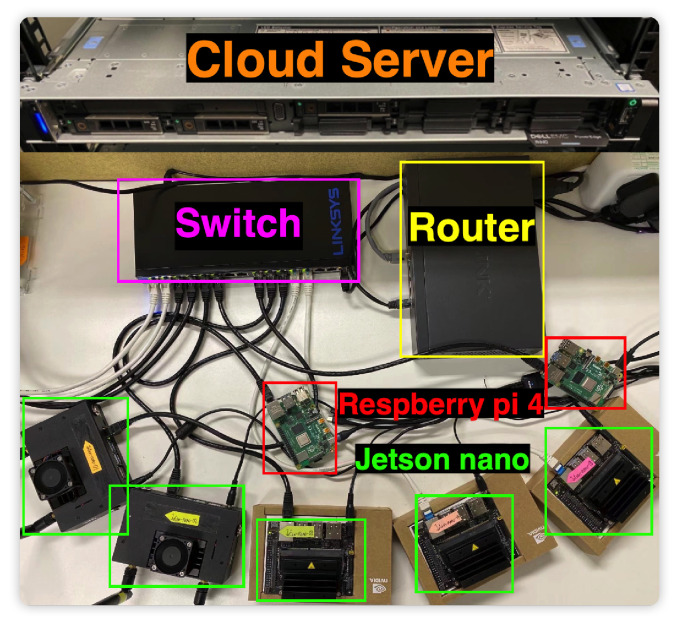
Experiment Devices.

We deploy all our system components, including the MS algorithm on real-world testbeds, as shown in Figure 4. In *FedDAS*, the cloud server is deployed on an Ubuntu server with 20 core (Intel(R) Xeon(R) Silver 4114 CPU @ 2.20 GHz), Gigabit Ethernet and 64 GB memory. The edge devices consist of five Jetson-Nanos (https://developer.nvidia.com/embedded/jetson-nano-developer-kit accessed on 18 August 2022) with 4GB RAM, 128-core Maxwell GPU, Gigabit Ethernet and ARM Cortex-A57 CPU each, and two Respberry Pi 4 models with 4GB RAM, Gigabit Ethernet and 1.5 GHz 64-bit quad-core ARM Cortex-A72 CPU. The networking devices consist of a switcher with Gigabit Ethernet and a router with 10 Gigabit Ethernet.

For the proof-of-concept, all of our experiments have been conducted on a widely adopted benchmark Cifar10 [42] in vanilla federated learning FedAvg [7] and we trained the experimental models for 140 communication rounds with 1 local epoch in total.

We present the comparison of our solution with three well-known neural network models in Table 1: (i) a manually designed, widely used and influential model ResNet [21]. In order to let the size of the model close to the model searched from our system, we used ResNet20 (20 layers ResNet); (ii) two models searched from NAS-201-Bench sorted by a single indicator. They were searched by maximising evaluation accuracy and minimising total training time to accuracy. Three models searched by our FedMSA system were defined by γ=0.1,0.2,0.8.

In addition, we let FedMSA filter out the model with less than 70% accuracy by setting α=70 in the configuration file. On the other hand, all models’ latency measurement experiments are in batches to easily distinguish the gaps in the different experiments’ results. The forward and backward propagation latency is measured base on batch size of the training set (64), and inference latency is measured by batch size of the test set (32).

To evaluate our model selection algorithm, we select a related study that is also trying to get the best model without training. Similar to FedMSA, NASWOT was proposed in [43], which is also based on the NAS-201-Bench search space. However, they did not consider training efficiency in the cross-device federated learning setting.

### 5.2. Results

We present a comprehensive investigation of the proposed FedMSA method on Cifar10 benchmark. We divided the experiment into three control groups, and the experimental results of our MS algorithm are reported in Table 1 from one Jetson nano and one Raspberry Pi 4.

In the first group, we use the selected model structure with a higher model performance by setting γ=0.1 for FedMSA, a model structure searched by NASWOTand NAS (Max Acc) is the model structure with the highest accuracy directly searched in the NAS-201-Bench search space. As we can clearly see that although the global test accuracy of NAS (Max Acc) is 1.3% more than FedMSA γ=0.1, it got two times more in FLOPs of the model, training time per round and all model latency metrics in client devices than FedMSA (γ=0.1). Particularly noteworthy are some metrics with large base values, such as training time per round and model performance in latency where more than twice those metrics can be catastrophic for the tasks sensitive to training efficiency or inference delay. Besides, the model searched by NASWOT performs similar to NAS (Max Acc) in training efficiency, and model latencies matrix. But, the accuracy of the model was reduced 6.42% performance.

In the second group, we set γ=0.2 for FedMSA to search a model structure and select a model structure as 20 layers ResNet, which is widely used and has good performance. FedMSA (γ=0.2) has clear advantages in the number of parameters and FLOPs, which are important for resource-constrain and network congestion devices. Although the metrics in model forward propagation and inference latency of ResNet20 perform better on Jetson nano, many tasks are not sensitive to this small gap due to the relatively small base value. However, although the accuracy of ResNet is more than 3.5%, the model back propagation latency of FedMSA (γ=0.2) is twice as high as on a ResNet20, and the base value of it is so large that it has a significant impact on the overall model training efficiency. Thus our algorithm gets a smaller model and a faster training speed by sacrificing some accuracy.

In the final group, we get a model structure of the same model size as the model with the smallest model size in the NAS-201-Bench search space by setting γ=0.8 for FedMSA. At the same time, these two models have a very intimate performance regarding model forward and backward propagation latency and inference latency. It is worth noting that the model structure selected by FedMSA (γ=0.8) has additional 4.3% accuracy over the model structure searched by NAS (Min $T_{train}$).

#### Training Time Complexity with Accuracy

**Figure 5:**
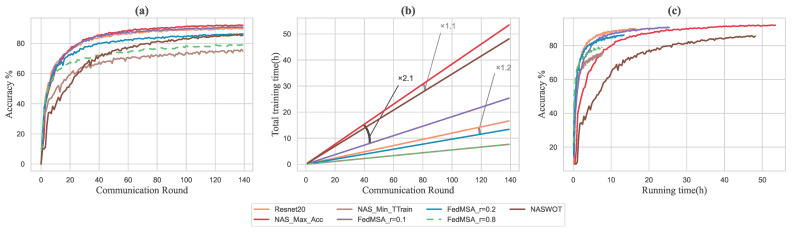
Experiments on Cifar10 under FedMSA system. (**a**) Validation accuracy of models, (**b**) Total training time spend in every communication round of models, (**c**) Changes in model accuracy over time.

The experimental result about training time complexity with accuracy is shown in Figure 5. For a distributed machine learning system, the training time complexity of the whole system decides the model training efficiency where a big training time complexity is unacceptable. The model structure search by NAS (Max Acc) has the highest accuracy in Figure 5a in 140 communication rounds, but the time spent on model training grows significantly rapidly in Figure 5b, and ended up being more than twice as high as the model structure searched by FedMSA (γ=0.1). In the early stage of Figure 5c, the accuracy achieved of NAS (Max Acc) is much less than others. Besides, the model structure searched by FedMSA (γ=0.1) can achieve similar accuracy with less training time growth rate, and it can be observed that through the complete training time of FedMSA (γ=0.1), it’s accuracy has always been significantly better than the NAS (Max Acc) in Figure 5c.

Furthermore, although FedMSA (γ=0.2) has less 3.5% accuracy than ResNet20 in Figure 5a, it has lower training time growth rate and finishes training earlier than ResNet20 in Figure 5b,c. Furthermore, although the two models searched by FedMSA (γ=0.8) and NAS (Min Ttrain) have the same total training time and growth rate in Figure 5b, the FedMSA (γ=0.8) has better performance during the whole training period with additional 4.2% accuracy than NAS(Min Ttrain) in Figure 5a,c.

## 6. Conclusions and Future Work

In this paper, we proposed FedMSA, a model selection and fast adaptation system which reduces the complexity of FL system deployment by providing automation of adaptation and deployment of training tasks as microservices in a FL life-cycle along with an optimal model selection algorithm. The evaluation shows that the model structure searched by the proposed MS algorithm consistently outperforms the model selected directly by one factor and the widely used model structure of other studies. Meanwhile, the results show that without a model selection algorithm, FL developers may end up selecting a model with more training time complexity and less accuracy compared with the model searched by a model selection algorithm from NAS search space under the same conditions.

As for future work, since our current model selection algorithm relies on the metrics of NAS-201-Bench, we will upgrade our algorithm to support more metrics of both models and distributed systems and extensively adapt our algorithm to other NAS. On the other hand, we will improve the system’s ability in finer tuning and adapting the result of model selection to improve the match between the results and the developer’s expectation by providing further configured parameters for developers or applying recommendation machine learning model.

## Figures and Tables

**Table 1:** Result Summary Total Communication Round 140.

Model	Numbers of Parameters (M)	FLOPs (M)	Training Time (m)/Round	Forward Propagation Latency (ms)	Back Propagation Latency (ms)	Model Inference Latency (ms)	Global Test Accuracy	Global Test Loss
**NAS(Max Acc)**	0.86 (×2.5)	15.61 (×2.6)	22.95 (×2.1)	2579.47 ^1^ (×2.1) 135.42 ^2^ (×1.9)	4889.23 ^1^ (×2.4) 160.49 ^2^ (×2.2)	2371.46 ^1^ (×2) 105.91 ^2^ (×1.7)	**92.05%**	**0.247**
**NASWOT**	0.46 (×1.35)	8.3 (×1.37)	20.78 (×1.92)	2681.47 ^1^ (×2.16) 148.65 ^2^ (×2)	3577.14 ^1^ (×1.78) 163.89 ^2^ (×2.30)	2438.59 ^1^ (×2.07) 105.03 ^2^ (×1.68)	85.63% (−6.42%)	0.435 (+0.188)
**FedMSA (γ=0.1)**	**0.34**	**6.06**	**10.8**	**1241.79 ^1^** **72.31 ^2^**	**2004.17 ^1^** **71.35 ^2^**	**1175.28 ^1^** **62.66 ^2^**	90.72% (−1.3%)	0.278 (−)
**RestNet20**	0.27 (×2.7)	5.16 (×3.3)	7.16 (×1.2)	**722.45 ^1^** **29.55 ^2^**	1560.13 ^1^ (×2) **41.21 ^2^**	**675.93 ^1^** **22.48 ^2^**	**89.77%**	**0.308**
**FedMSA (γ=0.2)**	**0.1**	**1.56**	**5.73**	754.07 ^1^ (−) 59.35 ^2^ (×2)	**779.78 ^1^** 47.86 ^2^ (×1.2)	695.81 ^1^ (−) 48.84 ^2^ (×2.2)	86.21% (−3.5%)	0.424 (+0.116)
**NAS (Min $T_{\mathrm{train}}$)**	**0.07**	**1**	**3.29**	410.5 ^1^ (−) 35.53 ^2^ (−)	395.29 ^1^ (−) **21.78** ^2^	393.10 ^1^ (−) 31.20 ^2^ (−)	74.9% (−4.2%)	0.743 (+0.11)
**FedMSA (γ=0.8)**	**0.07**	**1**	**3.29**	**409.03 ^1^** **35.04 ^2^**	**388.13 ^1^** 22.50 ^2^ (−)	**389.31 ^1^** **30.82 ^2^**	**79.10%**	**0.633**

^1^ From Raspberry Pi 4; ^2^ From Jetson nano.

## Data Availability

Not applicable.

## Abbreviations

The following abbreviations are used in this manuscript:

**Symbol **	**Description**
γ	User training efficiency concern rate
$T_{train}$	Total training time of the model to accuracy
$E_{train}$	Training efficiency of the model
$Acc_{eval}$	Expected final evaluation accuracy of the model
$Loss_{eval}$	Expected final evaluation loss of the model
Lat	The model inference latency in the hardware
$F_{model}$	The FLOPs of the model
*U*	Bandwidth utility of a device
*B*	Bandwidth of a device
$N_{params}$	Number of parameters of the model
*P*	All clients platform architecture information
*M*	All metrics of the model
$M_{c}$	All cleaned metrics of the model
$M_{n}$	All normalized metrics of the model
$M_{s}$	All metrics of the model with score
$M_{r}$	All ranked model base on the score
*X*	A set of model structures
*x*	A model structure
$S_{bit}$	Storage bit of developing framework
*C*	all clients of FL
